# Towards an Understanding of the Molecular Basis of Nickel Hyperaccumulation in Plants

**DOI:** 10.3390/plants8010011

**Published:** 2019-01-04

**Authors:** Llewelyn van der Pas, Robert A. Ingle

**Affiliations:** Department of Molecular and Cell Biology, University of Cape Town, Rondebosch 7701, South Africa; vpslle001@myuct.ac.za

**Keywords:** nickel, hyperaccumulation, serpentine, RNA-Seq, IREG, ferroportin, ZIP, histidine

## Abstract

Metal hyperaccumulation is a rare and fascinating phenomenon, whereby plants actively accumulate high concentrations of metal ions in their above-ground tissues. Enhanced uptake and root-to-shoot translocation of specific metal ions coupled with an increased capacity for detoxification and sequestration of these ions are thought to constitute the physiological basis of the hyperaccumulation phenotype. Nickel hyperaccumulators were the first to be discovered and are the most numerous, accounting for some seventy-five percent of all known hyperaccumulators. However, our understanding of the molecular basis of the physiological processes underpinning Ni hyperaccumulation has lagged behind that of Zn and Cd hyperaccumulation, in large part due to a lack of genomic resources for Ni hyperaccumulators. The advent of RNA-Seq technology, which allows both transcriptome assembly and profiling of global gene expression without the need for a reference genome, has offered a new route for the analysis of Ni hyperaccumulators, and several such studies have recently been reported. Here we review the current state of our understanding of the molecular basis of Ni hyperaccumulation in plants, with an emphasis on insights gained from recent RNA-Seq experiments, highlight commonalities and differences between Ni hyperaccumulators, and suggest potential future avenues of research in this field.

## 1. Introduction

Nickel is the most recent element to be classified as essential for plant growth [1], and to date, its only known biochemical function in plants is in the active site of the enzyme urease, which contains a bi-nickel center [2]. Presumably reflecting this, the requirement of plants for Ni is extremely low, and Ni deficiency, correspondingly rare. Higher plants typically contain Ni concentrations in the range of 0.5–10 mg kg^−1^ DW [3], and concentrations in excess of 10–50 mg kg^−1^ DW (depending on the plant species concerned) are associated with Ni toxicity effects [4]. These can include inhibition of photosynthesis, nitrogen assimilation, mitosis, and enzyme activity as well as DNA damage and the generation of reactive oxygen species [3,4]. While the majority of plants attempt to exclude excess Ni from their photosynthetically active tissues [5], a small group of plants known as Ni hyperaccumulators, actively accumulate Ni to concentrations in excess of 1000 mg kg^−1^ DW in their shoot tissues with no apparent toxicity effects [6].

Several hypotheses have been put forward to explain the adaptive function of metal hyperaccumulation in plants, of which the “elemental defence” hypothesis is most favored. This hypothesis, formulated by Boyd and Martens [7], proposes that the elevated concentrations of sequestered metal ions protect hyperaccumulators against attack by herbivores and pathogens. Feeding choice experiments carried out on several Ni hyperaccumulators have provided support for this hypothesis by demonstrating that herbivores opt to feed on leaf material with low Ni contents and that their fitness is reduced when forced to consume material with high Ni contents [8,9,10]. Ni hyperaccumulation has also been shown to reduce susceptibility to both bacterial and fungal pathogens in *Streptanthus polygaloides* [11], though it was associated with increased susceptibility of this species to Turnip mosaic virus [12]. Interestingly, the elemental defense provided by Ni has recently been shown to extend to the seeds produced by hyperaccumulators. Mortality rates of the generalist seed herbivore *Tribolium confusum* were significantly higher when fed seeds of *S. polygaloides* (containing 300 µg Ni g^−1^ DW) versus *Streptanthus insignis* (5 µg Ni g^−1^ DW), with an artificial diet experiment confirming that Ni concentrations > 240 µg Ni g^−1^ DW are toxic to this species [13].

There are currently 721 plant species in the global hyperaccumulator database, of which 532 are listed as Ni hyperaccumulators [14]. The taxonomic distribution of these species indicates that Ni hyperaccumulation has evolved multiple times. That said, 340 Ni hyperaccumulator species are found in just five families; Phyllanthaceae (118 species), Brassicaceae (87 species, 61 from the genus *Alyssum* in SE Europe and the Middle East), Cunoniaceae (48 species, all from New Caledonia), Asteraceae (45 species) and Euphorbiaceae (42 species, predominantly from Cuba). Since the publication of this database, several new Ni hyperaccumulators have been identified including *Senecio conrathii* from South Africa [15] and *Phyllanthus rufuschaneyi* from Borneo [16]. The preponderance of Ni hyperaccumulators among known metal hyperaccumulating plants likely reflects the fact that the serpentine soils with which they are associated are the most widespread metalliferous soils on a global scale [17]. Serpentine soils are derived from ultramafic rocks, and typically display a high Mg to Ca ratio, low levels of macronutrients and high levels of Ni, Co, and Cr [18,19]. The ultramafic regions of Turkey, Cuba, and New Caledonia, in particular, are regarded as “hotspots” of Ni hyperaccumulator biodiversity (see Reference [17] for a recent review of the ecology and distribution of hyperaccumulators).

The majority of Ni hyperaccumulators are restricted to serpentine soils (so-called obligate hyperaccumulators) where they can form almost pure species stands [17]. In contrast, the ranges of facultative hyperaccumulators extend outside of ultramafic outcrops [20]. While most facultative hyperaccumulators accumulate Ni whenever found on serpentine soils, some exceptions have been reported. For example, *Senecio coronatus* (Asteraceae) and *Alyssum sibiricum* (Brassicaceae) accumulate Ni at some but not all serpentine sites within their ranges [21,22]. Substantial intraspecific variation in Ni content can result from environmental factors, as is the case for *Pimelea leptospermoides* where shoot Ni contents ranging from 13 to 2873 mg kg^−1^ DW have been attributed to variation in total soil Ni content and pH [23]. However, in *S. coronatus,* this phenotypic variation has a genetic basis; plants from hyperaccumulator and non-accumulator populations have different root ultra-structures, and the accumulation phenotype of a given population persists when the plants are grown on a common soil substrate [24,25].

**Figure 1:**
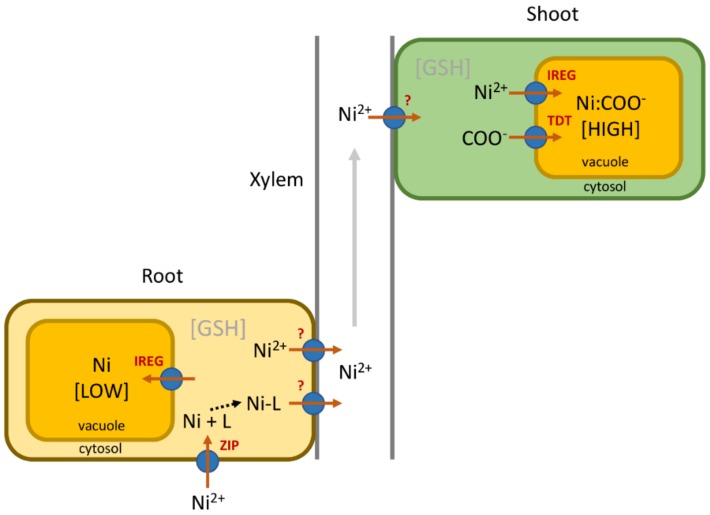
A general model for nickel hyperaccumulation in plants. Enhanced uptake of the Ni^2+^ cation from soil may be driven by high constitutive expression of poorly selective ZRT-IRT-like (ZIP) transporters responsible for Fe or Zn uptake. Chelation of Ni by an appropriate ligand (L) in the root cytosol is thought to prevent cytotoxicity. The identity and universality of the ligands used are an ongoing topic of debate, but histidine or nicotianamine perform this role in some Brassicaceae Ni hyperaccumulators. Formation of a Ni-ligand (Ni-L) complex may also impede the vacuolar sequestration of Ni in root tissues by tonoplast localized iron-regulated/ferroportin (IREG/FPN) transporters. Whether Ni is loaded into the xylem as the free cation or as a Ni-ligand complex is unclear, and the transporter(s) involved have not been identified, but the majority of Ni is present as the free cation in xylem sap. The transporter(s) involved in xylem unloading into shoot cells is also unknown. Ni is accumulated primarily in the shoot epidermis in most species, with the vacuole the major subcellular site of Ni sequestration. Constitutively high expression of IREG/FPN transporters has been reported in Ni hyperaccumulators versus related non-accumulators across four families, and two of these transporters have been shown to drive vacuolar sequestration of Ni. In the vacuole Ni is complexed by carboxylic acids (COO^−^), with Ni-citrate or Ni-malate the predominant complexes identified to date. Ni hyperaccumulating *Senecio coronatus* plants display greatly increased expression of the tonoplast dicarboxylate transporter (TDT) compared to non-accumulators. Ni hyperaccumulators have enhanced capacity for the detoxification of reactive oxygen species, which may involve elevated concentrations of glutathione (GSH), flavonoids or increased activities of anti-oxidant enzymes.

Like other metal hyperaccumulators, Ni hyperaccumulating plants display enhanced uptake, root to shoot translocation, and ability to detoxify and sequester Ni than non-accumulator species [26] (Figure 1). However, the molecular basis of these processes, and notably the transporters involved, is not well understood. In part, this is because the majority of research efforts aiming to uncover the molecular basis of metal hyperaccumulation have been directed at two Zn/Cd accumulators, *Arabidopsis halleri* and *Noccaea caerulescens* [20]. This is a consequence of their relatively recent divergence from *Arabidopsis thaliana*, which allowed the use of the genomic tools developed for this model plant. Expression profiling experiments have shown that these two hyperaccumulators have constitutively elevated expression of genes involved in the uptake, chelation, and xylem loading of Zn/Cd in comparison to related non-accumulators [27,28,29]. While it has been assumed that the same must hold true in Ni hyperaccumulators, the lack of genomic resources for these species has made this difficult to determine.

This situation has changed recently with the advent of RNA-Seq technology which allows the de novo assembly of transcriptomes and quantification of transcript abundance in the absence of any prior sequence information, and to date, four such studies have been published on Ni hyperaccumulators. Merlot et al. [30] carried out de novo transcriptome assembly of the New Caledonian hyperaccumulator *Psychotria gabriellae*, while Halimaa et al. [31] performed a comparative analysis of gene expression in the roots of three accessions of *N. caerulescens*, including one from a Ni hyperaccumulating serpentine population. While representing significant milestones in the study of Ni hyperaccumulation, both studies had limitations. The first was restricted to a single species meaning that the gene expression values derived from it cannot readily be used to identify genes expressed at higher levels in Ni hyperaccumulators versus non-accumulators. Interpretation of the second study is complicated by the fact that the three *N. caerulescens* populations also vary in their capacity to accumulate Zn/Cd, are geographically distant and grow on very different soils. Subsequently, two comparative RNA-Seq studies have been reported. Meier et al. [24] performed a comparative RNA-Seq analysis of Ni hyperaccumulating and non-accumulating serpentine populations of *S. coronatus* to identify candidate genes that may underpin the Ni hyperaccumulation phenotype. A large-scale RNA-Seq study comparing seven pairs of related Ni hyperaccumulating and non-accumulating species across five families (Brassicaceae, Rubiaceae, Cunoniaceae, Salicaceae and Euphorbiaceae) from Cuba, New Caledonia and France has recently been reported [32]. In addition to facilitating the identification of changes in gene expression that are common across multiple independent evolutionary origins of Ni hyperaccumulation, this study reported the first gene knockdown experiment in a Ni hyperaccumulator species. Here we review what is currently known about the molecular basis of the physiological processes underlying Ni hyperaccumulation in plants, with an emphasis on newly published results, and highlight the main challenges ahead and questions still to be answered.

## 2. Ni Uptake

To date, there is no evidence to suggest the existence of a Ni-specific transporter in plants for the uptake of this cation from soil. Instead, Ni uptake by roots appears to be catalyzed by poorly selective cation transporters, notably members of the ZRT/IRT-like (ZIP) family [33,34]. In *A. thaliana*, IRT1 is expressed in the root epidermis in response to Fe deficiency and is the primary route of Fe uptake in strategy I plants. However, IRT1 has a broad substrate specificity towards divalent cations and also transports Zn, Co, Cd, Mn, and Ni [35,36]. IRT1 is thought to be the primary route for Ni uptake in *A. thaliana*, at least under Fe limiting conditions. In wild-type plants, root Ni concentrations increased from one μmol g^−1^ FW under Fe-replete conditions to three μmol g^−1^ FW under Fe-deficiency, but remained unchanged in *irt1* mutants [32]. A more modest increase (less than 40%) in root Ni concentrations has also been reported under Zn-deficient conditions in *A. thaliana*, though the transporter(s) involved in this uptake process are unknown. The root Zn transporter ZIP3 is not involved as a *zip3* mutant actually accumulated higher levels of Ni under Zn-deficiency than did wild-type plants [33].

In line with the hypothesis that Ni uptake by roots occurs via non-selective transporters, inhibition of Ni accumulation by Zn and Fe has been reported in Ni hyperaccumulators. For example, supplementation of hydroponic media with equimolar Ni and Zn concentrations led to a significant reduction in shoot Ni content in the Zn/Ni hyperaccumulators *Noccaea pindicum* and *Noccaea alpinum* var. *sylvium* compared to plants treated with Ni only, while no inhibitory effect of Ni on Zn accumulation was observed [37]. Similarly, the presence of equimolar Ni and Zn concentrations had no effect on Zn uptake but resulted in an 80 to 90% reduction in Ni uptake in a serpentine population of *N. caerulescens* [38]. Such data suggest that Ni uptake in the *Noccaea* Ni hyperaccumulators is mediated via one or more Zn transporters.

In contrast, a recent study of Ni uptake kinetics in two *Alyssum* Ni hyperaccumulators, *Alyssum inflatum* and *Alyssum bracteatum*, found no evidence for inhibition of Ni uptake by equimolar concentrations of Zn [39]. Instead, in *A. bracteatum*, Ni uptake was modulated by the concentration of Fe present in hydroponic media. Plants grown in the absence of added Fe for one week displayed a higher V_max_ for Ni uptake, while those exposed to excess Fe had a higher K_m_ for Ni uptake [39]. These results are consistent with Ni uptake via an Fe-deficiency induced Fe transporter, such as IRT1, in this species. In stark contrast, the kinetics of Ni uptake in *A. inflatum* were unaffected by Fe concentration in the nutrient solution (while those of Fe were not) suggesting that Ni uptake does not occur via a Fe transporter in this species [39]. A subsequent study employing the Ca channel inhibitor verapamil lead to a significant decrease in Ni uptake in both species [40] which the authors suggest indicates that these may be a major route of Ni uptake. However, it is not possible to rule out pleiotrophic effects of verapamil on Ni uptake, and further experimental work is required to test this hypothesis.

Increased expression of several members of the ZIP family has been reported in two comparative RNA-seq studies of Ni hyperaccumulators. *S. coronatus* Ni hyperaccumulators display high expression of a *ZIP* transporter, putatively annotated as *IRT1* based on phylogenetic analysis, in their root tissues while non-accumulating serpentine plants do not [24]. Increased expression of transcripts putatively annotated as *IRT1* and *ZIP10* has also been reported in roots of serpentine versus non-serpentine populations of *N. caerulescens* [31]. However, annotation of ZIP transporters based solely on sequence similarity to *A. thaliana* ZIPs is problematic due to the extensive gene duplication that has occurred in the ZIP family. For example, in *A. thaliana* IRT1 is one of five paralogues which share high sequence similarity but have different functions.

## 3. Chelation and Xylem Loading of Ni

Chelation of Ni ions in the cytosol by ligands is thought to be an important tolerance mechanism by which hyperaccumulators prevent metal toxicity and growth impairment [41]. However, the nature of the ligands involved, and their relative importance remains a matter of some debate. The amino acid histidine (His) has been implicated in the tolerance and root-to-shoot transport of Ni in several hyperaccumulators from the Brassicaceae. *Noccaea goesingense* and *Alyssum lesbiacum* display constitutively elevated levels of free His in their root tissues compared to related non-accumulators [42,43], which in *A. lesbiacum*, at least, is due to elevated expression of the first enzyme in the His biosynthetic pathway ATP-phosphoribosyl transferase (ATP-PRT) [42]. *A. thaliana* plants over-expressing ATP-PRT display both elevated His contents and tolerance to Ni [42,44,45].

Nicotianamine (NA), which is synthesized from three molecules of S-adenosylmethionine, has also been implicated as a Ni chelator in the genus *Noccaea*. A Ni-NA complex has been identified in *N. caerulescens* [46], and a strong positive correlation between shoot Ni and NA contents across seven species was observed by Callahan et al. [47]. Over-expression of NA synthase in *A. thaliana* and tobacco results in increased NA levels and Ni tolerance [48]. However, as is the case for His, there is little evidence to date to suggest that NA is a significant chelator of Ni outside the Brassicaceae.

The identity of the transport protein(s) responsible for the xylem loading of Ni remains elusive. Exogenous Ni elicits a dose-dependent increase in xylem His levels in several *Alyssum* hyperaccumulators [49,50] suggesting that His might also be involved in xylem loading of Ni. However, this does not occur in the non-accumulator *Alyssum montanum* [50] nor in transgenic *A. thaliana* plants with elevated free His levels due to over-expression of ATP-PRT [42]. This has been interpreted as indicating that Ni hyperaccumulators may possess a transport protein, lacking in other plant species, that loads the Ni-His complex into the xylem, though no such transporter has been identified to date. It is also clear that the majority of Ni in the xylem exists as the free cation in the *Alyssum* Ni hyperaccumulators [50,51], and that xylem Ni concentrations are an order of magnitude higher than those of His [51]. As His strongly promotes Ni uptake by root-derived tonoplast vesicles of the non-accumulator *Noccaea arvense* but has a modest repressive effect on vesicles from *N. caerulescens* [52], it has been suggested that His could promote root-to-shoot translocation of Ni by reducing vacuolar sequestration of Ni in root tissues of hyperaccumulators [52], though the mechanism by which this might occur is unknown. The observation that root to shoot translocation of Ni was enhanced in both *A. inflatum* and *A. bracteatum* under conditions of Fe deficiency suggests that xylem loading of Ni may be mediated by the same transporter(s) responsible for the loading of Fe [37], which is thought to be as the free Fe^3+^ cation. The identity of this transporter is unknown, but IREG1 a member of the iron-regulated/ferroportin (IREG/FPN) family which localizes to the plasma membrane and displays vasculature-specific expression has been proposed as a candidate [53], and might conceivably also transport Ni.

## 4. Vacuolar Sequestration of Nickel

The shoot epidermis is the primary site of Ni deposition in the majority of Ni hyperaccumulators studied to date [54,55]. The transporters responsible for the unloading of Ni (and Fe) from xylem into shoot cells are unknown but may include members of the ZIP family [56]. Interestingly, the *ZIP* transporter identified from *S. coronatus* displays differential expression in roots and shoots of Ni hyperaccumulators versus non-accumulator plants and is most highly expressed in shoots [24]. At a subcellular level, the vacuole is the primary site of Ni storage in shoot cells [55,57], and vacuolar ATPase-dependent Ni/proton antiport activity has been demonstrated in vacuoles derived from shoot cells of *A. lesbiacum* [58].

In *A. thaliana*, IREG2, another member of the IREG/FPN family, mediates the vacuolar sequestration of Ni inadvertently taken up by IRT1 in roots, as evidenced by the reduced Ni tolerance of *ireg2* mutants under Fe-deficiency conditions [36]. There is increasing evidence to suggest that vacuolar uptake of Ni may also be mediated by IREG/FPN transporters in Ni hyperaccumulators. Merlot et al. [30] identified a vacuolar-targeted IREG (PgIREG1) from *P. gabriellae* and demonstrated using RT-qPCR that it is expressed at higher levels in this species than in the non-accumulator *Psychotria semperflorens*. Heterologous expression of *PgIREG1* complemented the hypersensitivity of root growth to Ni and reduced root Ni content phenotypes of the *A. thaliana ireg2* mutant, and a PgIREG1:GFP fusion protein localized to the vacuolar membrane [30]. While *PgIREG1* is a functional orthologue of *IREG2*, its pattern of expression differs markedly; in *A. thaliana IREG2* expression is restricted to the root and occurs only under Fe deficiency, while in *P. gabriellae* high constitutive expression occurs in both root and shoot tissues [30]. A similar pattern of expression was observed for the putative *IREG* homologue identified in *S. coronatus* Ni hyperaccumulators [24].

The recent cross-species RNA-Seq study performed by de la Torre et al. [32] has revealed that elevated expression of *IREG* transporters in shoot tissues is a common feature of Ni hyperaccumulators from the Brassicaceae (*N. caerulescens*), Euphorbiaceae (*Leuococroton havanensis*) and Rubiaceae from both New Caledonia (*P. gabriallae*) and Cuba (*Psychotria grandis* and *Psychotria costivenia*) compared to related non-accumulators. Notably, this study also reported the first gene silencing experiments to be carried out in a Ni hyperaccumulator. Roots of *N. caerulescens* were transformed with *Rhizobium rhizogenes*, and gene knockdown of *IREG* achieved through the production of artificial miRNA. The resulting decrease in root Ni contents, together with the tonoplast localization of the transporter, support a role for *IREG* in the vacuolar sequestration of Ni [32].

While carboxylic acids, such as citrate and malate, have low stability constants with Ni at cytosolic pH, they are the predominant ligands for Ni in the acidic environment of the vacuole [59]. The carboxylic acid utilized appears to vary between Ni hyperaccumulators. High levels of citrate and the presence of a Ni-citrate complex were reported from shoot tissues of several New Caledonian Ni hyperaccumulators including *Pycnandra acuminata* [60,61]. In contrast, malate appears to be the primary ligand for Ni in shoot tissues of the Brassicaceae Ni hyperaccumulators [62,63]. Both Ni-citrate and Ni-malate complexes have been identified in the South African Asteraceae hyperaccumulators *Berkheya coddii* and *S. coronatus* [64], and Ni hyperaccumulating *S. coronatus* plants display greatly elevated expression of the tonoplast dicarboxylate transporter in their shoot tissues in comparison to non-accumulators [24].

## 5. Protection against Oxidative Damage

As it is not redox active, Ni cannot generate reactive oxygen species (ROS) directly via electron transfer but can disrupt the balance between formation and destruction of ROS during metabolism. This is thought to occur via direct binding of Ni to proteins or by displacement of essential cations from binding sites thereby leading to oxidative stress [65]. Exposure to Ni has been shown to result in increased ROS levels in Ni hyperaccumulators. For example H_2_O_2_ levels increased 3.6-fold in hairy root cultures of *Alyssum bertolonii* in response to 25 ppm Ni [66], while dichlorofluorescein-based imaging revealed that ROS levels increased in roots of both *Alyssum murale* (hyperaccumulator) and *A. montanum* (non-accumulator) when transferred from hydroponic media lacking added Ni to media containing 80 μM Ni [67]. Such experiments indicate that Ni hyperaccumulators do not have the ability to prevent Ni-induced ROS formation from occurring, and instead, it seems likely that they possess enhanced ROS detoxification capacity.

Whether Ni hyperaccumulators possess constitutively elevated anti-oxidant enzyme activities to deal with Ni-induced ROS relative to non-accumulators is debatable. Some studies have suggested that this is the case, for example, super-oxide dismutase (SOD) and catalase activities were determined to be 2.4 and 500 times greater respectively in *A. bertolonii* versus tobacco hairy root cultures [66]. In contrast, other studies have found little or no difference. For example, in a study comparing *A. inflatum*, *A. bracteatum* (Ni hyperaccumulators) and *Alyssum saxatile* (non-accumulator), SOD activities were less than two-fold higher in roots of the hyperaccumulators and identical in shoot tissues [68]. There is also no evidence from RNA-Seq experiments to suggest that mRNA levels of these anti-oxidant enzymes are elevated in hyperaccumulators versus non-accumulators [24,32]. It is apparent that the activity of anti-oxidant enzymes can be overcome by high concentrations of Ni, as demonstrated by the decline in SOD and catalase activities observed in *A. markgrafii* when grown hydroponically in the presence of 0.5 mM Ni, correlating with reduced biomass production in this species [69].

In addition to enzymatic anti-oxidants, plants also contain non-enzymatic anti-oxidants that scavenge ROS including glutathione (GSH) and ascorbate. A strong positive correlation between shoot Ni and GSH contents has been demonstrated in the genus *Noccaea* [70]. This has been attributed to elevated activity of serine acetyltransferase (SAT) in Ni hyperaccumulating members of this genus, which is required for the production of cysteine (Cys), a component of GSH. *A. thaliana* plants over-expressing SAT from *N. goesingense* contain elevated levels of Cys and GSH and display increased Ni tolerance [70]. The regeneration of reduced GSH from oxidized glutathione disulphide (GSSG) is catalyzed by the enzyme glutathione reductase, and *N. goesingense* has both an increased glutathione reductase (GR) activity and GSH:GSSG ratio in comparison to *A. thaliana* [70]. GR activities and GSH levels have also been shown to increase in response to Ni in the hyperaccumulators *A. inflatum*, *A. bracteatum*, and *A. markgrafii*, but this does not occur in the non-accumulator *A. saxatile* [68,69]. Together these data suggest that the increased size of the GSH pool, and the ability to maintain the GSH:GSSG ratio through the activity of GR may be an important component of Ni tolerance in hyperaccumulators, at least, in the Brassicaceae.

In the cross-species RNA-Seq experiments performed by de la Torre et al. [32], ten of the 33 clusters of orthologous genes more highly expressed in at least three of the hyperaccumulator versus non-accumulator pairwise species comparisons function in phenylpropanoid and flavonoid biosynthesis. Flavonoids have been suggested to function as scavengers of ROS generated during environmental stress, and their synthesis increases in plants under such conditions [71]. Support for this hypothesis has been provided by transgenic *A. thaliana* plants over-expressing *MYB12* which contain elevated levels of flavonoids, particularly anthocyanins, and display increased tolerance to oxidative and drought stress [72]. Whether ROS scavenging by flavonoids plays a role in Ni hyperaccumulators is unknown but is a worthwhile avenue for future research. Finally, Ni can cause DNA damage through direct oxidation of guanine residues, inhibition of DNA repair systems or by promoting the formation of ROS [73]. The observation that exogenous Ni caused DNA damage leading to reduced genomic integrity in *A. thaliana* but not in serpentine *N. caerulescens*, led to the suggestion that the capacity to maintain genome integrity in the presence of high levels of Ni is an important component of the hyperaccumulation phenotype [74]. In line with this hypothesis, *S. coronatus* hyperaccumulators have elevated expression of several genes involved in telomere maintenance, DNA repair and maintenance of repressive epigenetic marks in comparison to non-accumulators [24].

## 6. Conclusions and Potential Directions of Future Research

Ni hyperaccumulation has evolved multiple times in plants, but whether it has done so via the same route on each occasion is unknown. For some aspects of the Ni hyperaccumulation phenotype, there is evidence of convergent evolution, most notably in the high constitutive expression of *IREG*/*FPN* orthologues implicated in vacuolar sequestration of Ni in the shoot tissues of Ni hyperaccumulators from the Asteraceae, Brassicaceae, Euphorbiaceae, and Rubiaceae [24,30,32]. However other components, such as the role of His or NA as significant Ni-binding ligands, appear to be restricted to specific plant lineages. There is also evidence suggesting that the route by which Ni is taken up from the soil varies between hyperaccumulators within the same family, with Zn transporters implicated in *Noccaea* hyperaccumulators and Fe transporters in *Alyssum* hyperaccumulators [37,38,39]. It is possible that this implies that different ZIP transporters can be recruited during the evolution of Ni hyperaccumulation. Given the known role of the ZIP transporter IRT1 in Ni uptake in *A. thaliana* [34], increased expression of members of the ZIP family in roots of *N. caerulescens* and *S. coronatus* suggests that they might be involved in driving enhanced Ni uptake from the soil. However, the extensive expansion of the ZIP gene family in *A. thaliana* means that it is problematic to infer potential transport activities simply on the basis of sequence homology to *A. thaliana* ZIP transporters. For example, a putative IRT1 orthologue from barley was recently shown to transport Mn but not Fe [75]. Functional characterization of candidate Ni transport proteins to determine their actual substrate range is essential and could be accomplished through heterologous expression in yeast or by complementation of *A. thaliana* or yeast transporter mutants.

Better still would be to demonstrate that a candidate gene identified in a Ni hyperaccumulator is indeed required for Ni hypertolerance or accumulation in that species. This is essential if our understanding of the molecular basis of Ni hyperaccumulation is to progress beyond drawing correlations between mRNA or metabolite levels and Ni tolerance or accumulation in a hyperaccumulator species. To this end, the strategy of *R. rhizogenes* mediated transformation to effect gene silencing through artificial miRNA production recently used by de la Torre et al. [32] may be applicable to other Ni hyperaccumulators. Alternatively, CRISPR/Cas9-mediated mutation offers great potential to test gene function in Ni hyperaccumulators, and has recently been used in the Cd hyperaccumulator *Sedum plumbizincicola* to demonstrate that heavy metal ATPase 1 (HMA1) is required for Cd export from the chloroplast in order to prevent inhibition of photosynthesis [76].

Finally, what drives the elevated expression of the FPN/IREG and ZIP transporters implicated in Ni transport in Ni hyperaccumulators? Zn accumulation in both *N. caerulescens* and *A. halleri* results (at least in part) from elevated expression of the *HMA4* transporter responsible for xylem loading of Zn, leading to Zn deficiency in the root tissues of these species, and so to up-regulation of the transporters involved in Zn uptake [77,78]. In *A. thaliana*, *IRT1* and *IREG2* expression is regulated by the transcription factor FIT, which is itself activated in response to Fe deficiency [79]. *S. coronatus* hyperaccumulators display elevated expression of a putative *FIT* orthologue in comparison to non-accumulators, however this transcript was only detected in shoot tissues, while increased expression of the putative *IRT1* and *IREG* orthologues occurs in both roots and shoots [24]. Increased gene copy number and changes in promoter activity have been implicated in the increased *HMA4* expression observed in Zn hyperaccumulators [77,78]. Analysis of gene copy number and promoter DNA sequences of candidate Ni hyperaccumulation genes, coupled with yeast-one-hybrid screens to identify the transcription factors controlling their expression, will shed light on the regulation and evolution of this fascinating phenotype in plants.
